# Use of Fe_3_O_4_ Nanoparticles for Enhancement of Biosensor Response to the Herbicide 2,4-Dichlorophenoxyacetic Acid

**DOI:** 10.3390/s8095775

**Published:** 2008-09-18

**Authors:** Kee-Shyuan Loh, Yook Heng Lee, Ahmad Musa, Abdul Aziz Salmah, Ishak Zamri

**Affiliations:** 1 School of Chemical Sciences and Food Technology, Faculty of Science and Technology, University Kebangsaan Malaysia, Bangi, 43000, Selangor, Malaysia; E-mails: keeshyuan@yahoo.com; andong@ukm.my; 2 Biotechnology Research Centre, Malaysian Agriculture Research & Development Institute (MARDI), P.O. BOX 12301, GPO Kuala Lumpur, Malaysia; E-mails: salmahaa@mardi.gov.my; zamri@mardi.gov.my

**Keywords:** Screen-printer biosensor, alkaline phosphatase, Fe_3_O_4_ nanoparticles, 2, 4-dichlorophenoxyacetic acid

## Abstract

Magnetic nanoparticles of Fe_3_O_4_ were synthesized and characterized using transmission electron microscopy and X-ray diffraction. The Fe_3_O_4_ nanoparticles were found to have an average diameter of 5.48 ±1.37 nm. An electrochemical biosensor based on immobilized alkaline phosphatase (ALP) and Fe_3_O_4_ nanoparticles was studied. The amperometric biosensor was based on the reaction of ALP with the substrate ascorbic acid 2-phosphate (AA2P). The incorporation of the Fe_3_O_4_ nanoparticles together with ALP into a sol gel/chitosan biosensor membrane has led to the enhancement of the biosensor response, with an improved linear response range to the substrate AA2P (5-120 μM) and increased sensitivity. Using the inhibition property of the ALP, the biosensor was applied to the determination of the herbicide 2,4-dichlorophenoxyacetic acid (2,4-D). The use of Fe_3_O_4_ nanoparticles gives a two-fold improvement in the sensitivity towards 2,4-D, with a linear response range of 0.5-30 μgL^-1^. Exposure of the biosensor to other toxicants such as heavy metals demonstrated only slight interference from metals such as Hg^2+^, Cu^2+^, Ag^2+^ and Pb^2+^. The biosensor was shown to be useful for the determination of the herbicide 2, 4-D because good recovery of 95-100 percent was obtained, even though the analysis was performed in water samples with a complex matrix. Furthermore, the results from the analysis of 2,4-D in water samples using the biosensor correlated well with a HPLC method.

## 1. Introduction

Magnetic nanoparticles such as Fe_3_O_4_ nanoparticles have been widely used in the collection and separation of bioactive molecules, targeted drug delivery and biomedical applications. The many techniques of immobilization of biomolecules on the surface of magnetic nanoparticles have enabled the production of bioconjugates with magnetic properties and these substances are useful for the delivery and recovery of biomolecules in biomedical applications. Because of their submicron dimensions and special chemical and physical properties, Fe_3_O_4_ nanoparticles have been applied to the construction of biosensors based on DNA, proteins and enzymes with reported improved detection limits, sensitivity, and reduced response times. A very important property of this type of nanoparticles for electrochemical biosensors is their ability to provide a favourable microenvironment for biomolecules such as proteins to exchange electrons directly with an electrode [1], thus improving the sensitivity of electrochemical biosensors. Fe_3_O_4_ nanoparticles have been used for the construction of enzyme based biosensors involving laccase [2-4], tyrosinase [5-6], glucose oxidase [7-8] and horseradish peroxidase [9].

Production of many enzyme based biosensors using Fe_3_O_4_ nanoparticles involves complicated enzyme immobilization procedures via surface modification of the nanoparticles in order to prevent loss of the enzyme. The biological molecules are either bound directly to the nanoparticles via surface modification with amino groups or require cross-linking agents [2, 6, 7]. However, simple immobilization techniques for enzymes by entrapping them in a polymer membrane, which was doped with Fe_3_O_4_ nanoparticles were also reported [5, 8].

Because the herbicide 2,4-dichlorophenoxyacetic acid (2,4-D) is carcinogenic, teratogenic and estrogenic, its determination is an important analysis that can ensure both environmental and food safety. Many biosensors reported for the detection of 2,4-D were immunosensors [10-13]. Several of these immunosensors employed the enzyme alkaline phosphatase as a label [14-16]. Other non-antibody based biosensors for detection of 2,4-D were based on a bienzyme system of alkaline phosphatase-glucose oxidase [17] or a system of tyrosinase-microbe [18]. Some disadvantages of immunosensors are the need for the production of antibodies, which are generally not commercially available, via immunization of an animal, and also the labeling of the antibody with enzyme. These result in costly and complicated biosensor fabrication processes. Although immunosensors and other bienzyme based biosensors reported so far have demonstrated high sensitivity and specificity towards 2,4-D, these devices were complicated to fabricate and operate because of the many biomolecules that are involved.

In this work we report the use of the enzyme alkaline phosphatase (ALP) together with the Fe_3_O_4_ nanoparticles to construct an enzyme based biosensor for 2,4-D determination. The combination of ALP and Fe_3_O_4_ nanoparticles as a sensing device for 2,4-D so far has not been explored. The advantage of the biosensor in this work when compared to other reported biosensors was the simple fabrication procedure, which merely involved the entrapment of both ALP and Fe_3_O_4_ nanoparticles in a hybrid sol-gel/chitosan membrane deposited on top of a screen-printed carbon paste electrode to produce a disposable biosensor for 2,4-D detection. The main focus of this work was to investigate the effect of the Fe_3_O_4_ nanoparticles on the enzyme reaction and subsequently its influence on the inhibition by 2,4-D and several other toxic metals. To demonstrate the usefulness of the biosensor developed, water samples from rice fields were analysed for 2,4-D with the biosensor.

## 2. Results and Discussion

### 2.1 Characterization of the Fe_3_O_4_ magnetic nanoparticles

**Figure 1.**
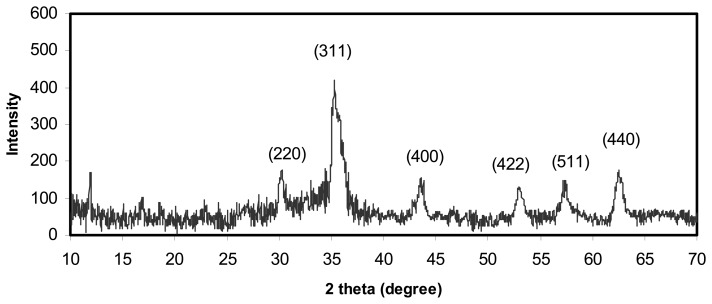
The X-ray diffraction pattern of a sample of Fe_3_O_4_ nanoparticles. The spinel diffraction pattern is confirmed from the *hkl* indices given.

The purity of the prepared Fe_3_O_4_ nanoparticles was examined using X-ray diffraction. The XRD pattern of the final powders is show in Figure 1. The XRD spectrum exhibited peaks corresponding to Fe_3_O_4_, marked with their indices (220), (311), (400), (422), (511) and (440), which is similar to that reported before for Fe_3_O_4_ nanoparticles [19, 30].

**Figure 2.**
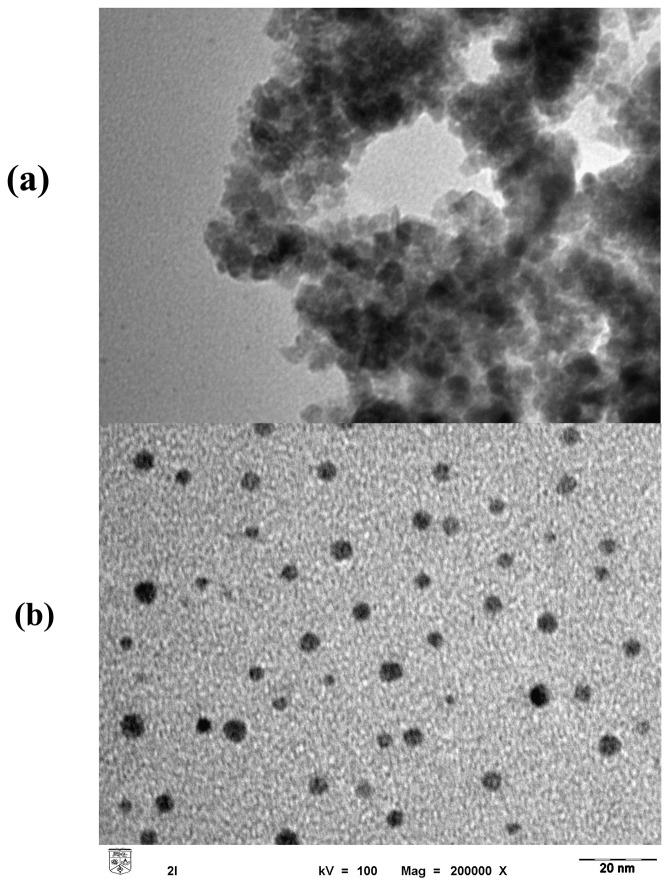
The transmission electron micrograph showing the size and distribution of Fe_3_O_4_ nanoparticles in ethanol solution (a) and ALP solution (b).

The morphology and structure of the synthesized Fe_3_O_4_ nanoparticles were examined using transmission electron micrography (Figure 2). It is clear that Fe_3_O_4_ nanoparticles were very fine, with an average diameter of 5.48 ±1.37 nm. The cluster-like structure of the particles (Figure 2a) may be the result of adsorption of molecules from the surroundings onto the surface to achieve inter-molecular force equilibrium [20]. However, when the enzyme ALP was mixed with the Fe_3_O_4_ nanoparticles, the cluster-like structure disappeared completely and the nanoparticles seemed to be separated (Figure 2b). The most possible explanation for this phenomenon is the adsorption of the protein (ALP) on the surface of nanoparticles, which reduced the magnetic moment [21] and diminished the inter-molecular forces exerted between the nanoparticles.

### 2.2 Optimum conditions for biosensor operation

The response of the biosensor is based on the current generated by the oxidation of ascorbic acid produced from the catalyzed hydrolysis of the substrate ascorbic acid 2-phosphate (AA2P) by ALP:

$$\text{Ascorbic acid}\;2-\text{phosphate}\overset{\text{ALP}}{\to }\text{Ascorbic acid}+{\text{H}}_{3}{\text{PO}}_{4}$$

$$\text{Ascorbic acid}\overset{\text{Oxidation}}{\to }\text{Dihydro ascorbic acid}+2{\text{H}}^{+}+2{\text{e}}^{-}$$

When Fe_3_O_4_ nanoparticles are present, the mediation of electron transfer to the electrode occurs via the following reactions:

$$\begin{array}{l} {\text{Fe}}_{3}{\text{O}}_{4}+8{\text{H}}^{+}+2{\text{e}}^{-}\to 3{\text{Fe}}^{2+}4{\text{H}}_{2}\text{O}\\ {\text{Fe}}^{2+}\to {\text{Fe}}^{3+}+{\text{e}}^{-} \end{array}$$

**Figure 3.**
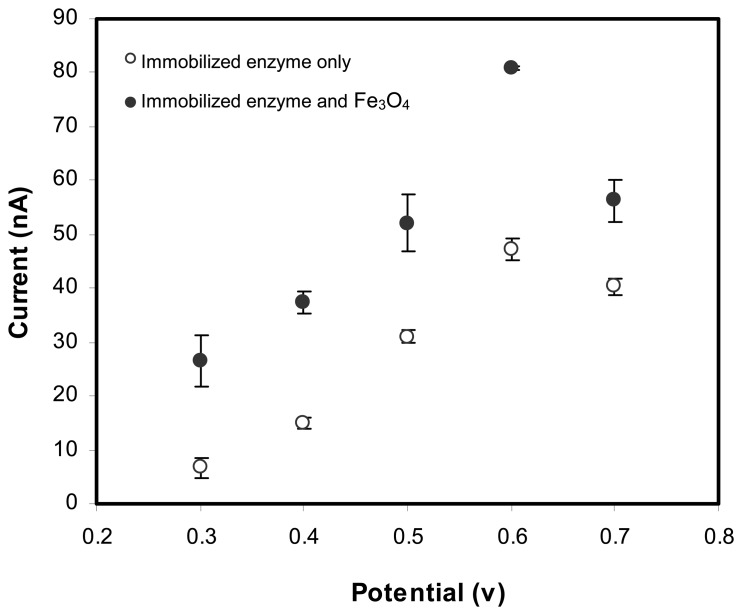
The hydrodynamic voltammograms (0.3 to 0.7 V) of the enzymic reaction between ALP and AA2P (80μM) for biosensor with ALP or ALP and Fe_3_O_4_ immobilized in a sol gel/chitosan membrane.

The hydrodynamic responses of the biosensors with immobilized ALP or ALP/Fe_3_O_4_ nanoparticles in Figure 3 show that the current increases gradually to reach a maximum of 0.6 V. Both of the immobilized ALP and ALP/Fe_3_O_4_ nanoparticles demonstrated the same response pattern where the current became steady at 0.7 V applied potential.

At 0.6 V, the current response of the biosensor with immobilized ALP and Fe_3_O_4_ was higher when compared to the biosensor without Fe_3_O_4_ nanoparticles immobilized. An approximately 50 % increase in current was observed. Alonso *et al.* [22] observed via cyclic voltammetric scans that reduction of Fe_3_O_4_ occurred to produce Fe(II), followed by oxidation to Fe(III) with the release of electrons. It is believed that this catalytic behaviour of Fe_3_O_4_ provides another pathway for electron transfer and improved the conductive properties. Lu and Chen have reported that for a glucose biosensor, an increase in response current after drop-coating a screen-printed carbon electrode with Fe_3_O_4_ nanoparticles [23]. This implies that the presence of Fe_3_O_4_ nanoparticles can provide a micro-environment favorable for promoting electron transfer to the electrode.

**Figure 4.**
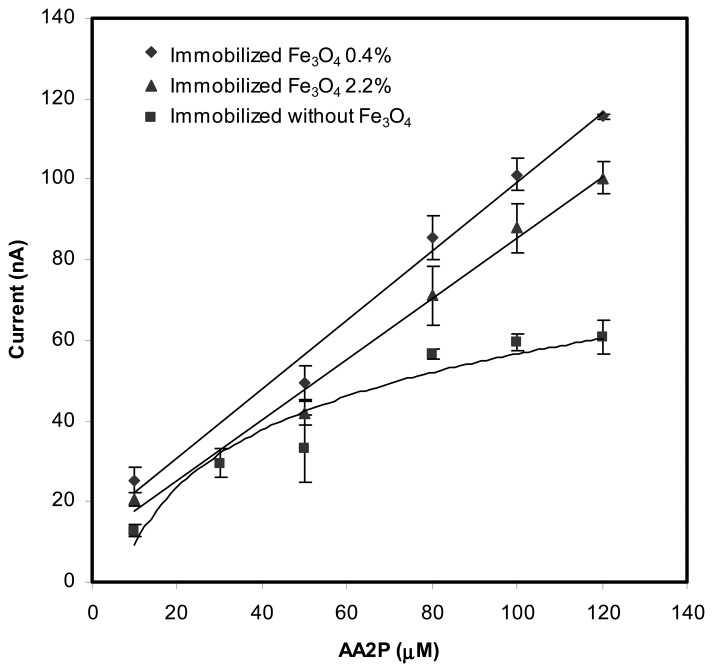
The response of biosensors with immobilized Fe_3_O_4_ (0.4 and 2.2 wt%) and without Fe_3_O_4_ in the presence of changing concentrations of AA2P substrate.

Figure 4 shows the behavior of the biosensors when different amounts of Fe_3_O_4_ nanoparticles, i.e. 0.4 and 2.2 wt% and ALP were incorporated into the biosensor membrane. For the same concentration of substrate, both of the biosensors with immobilized ALP and Fe_3_O_4_ (0.4 and 2.2 wt% of Fe_3_O_4_) gave higher current response when compared to the biosensor with no Fe_3_O_4_ nanoparticle incorporated.

The amount of Fe_3_O_4_ added into the membrane has an effect on the biosensor response. When compared with biosensors that have no Fe_3_O_4_ nanoparticles incorporated, the biosensors with Fe_3_O_4_ nanoparticles demonstrated wider linear response range to AA2P (Table 1). But higher amounts of Fe_3_O_4_, e.g. 2.2 wt% appears to reduce the sensitivity slightly by 0.125 nA μM^−1^ and the observed current response was also slightly smaller. This slight decrease in response may be ascribed to the excessive of nanoparticles in the membrane, which may obstruct the diffusion process of the reactions products towards the electrode surface [24]. Further addition of Fe_3_O_4_ to 3.5 wt% into the membrane caused the film to become brittle and cracked, hence no biosensor could be fabricated. For membrane with no Fe_3_O_4_ nanoparticle, the lowest response slope was observed. The amount of Fe_3_O_4_ nanoparticles also influenced the detection limits for AA2P. Membranes with 0.4 and 2.2 wt % of Fe_3_O_4_ demonstrated detection limits of 2.0 μM and 2.5 μM respectively. These detection limits were about 50% lower when compared to membrane with no nanoparticles (5.1 μM). Therefore, the optimum loading of nanoparticles should be close to 0.4 wt% and for further experiments, this amount of Fe_3_O_4_ nanoparticles was selected.

**Figure 5.**
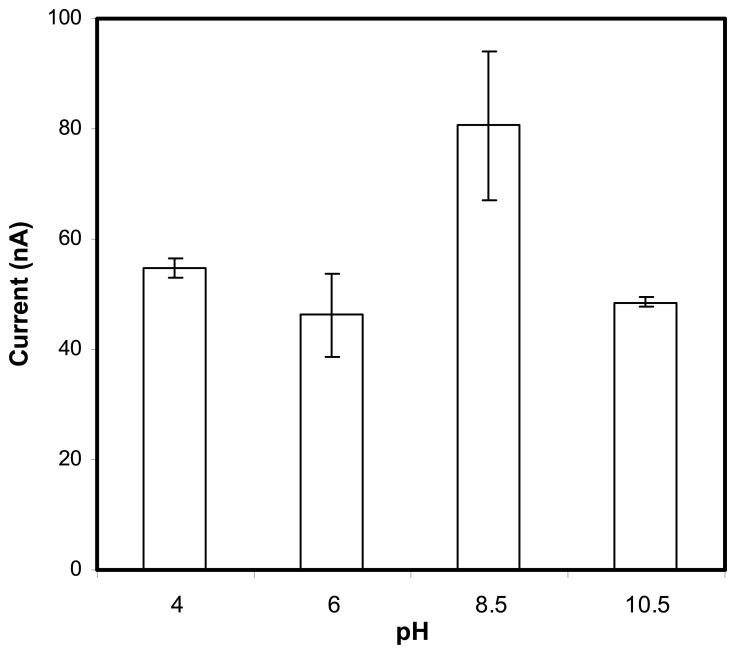
The effect of pH on the response of the biosensor containing Fe_3_O_4_ nanoparticles. The substrate concentration of AA2P for the study was fixed at 80 μM in 0.1 M Tris-HCl buffer with applied potential at 0.6 V versus Ag/AgCl reference.

The optimum operational pH of the biosensor was also examined. The enzyme ALP was reported to function best at pH 8-11 [25-27]. Figure 5 depicts the pH response of biosensor with ALP/Fe_3_O_4_ in the presence of a fixed concentration of the substrate AA2P. Clearly the highest current response was at pH 8.5 and this was within the optimum range of this enzyme. This indicates that the nanoparticles did not exert large effect on the enzymic reaction. Fe_3_O_4_ nanoparticles are generally considered to be biocompatible [28-29] and do not interfere with biological reactions. The optimum pH of 8.5 was used in further study on the determination of 2,4-D.

### 2.2. Determination of 2,4-D and heavy metals with biosensors

**Figure 6.**
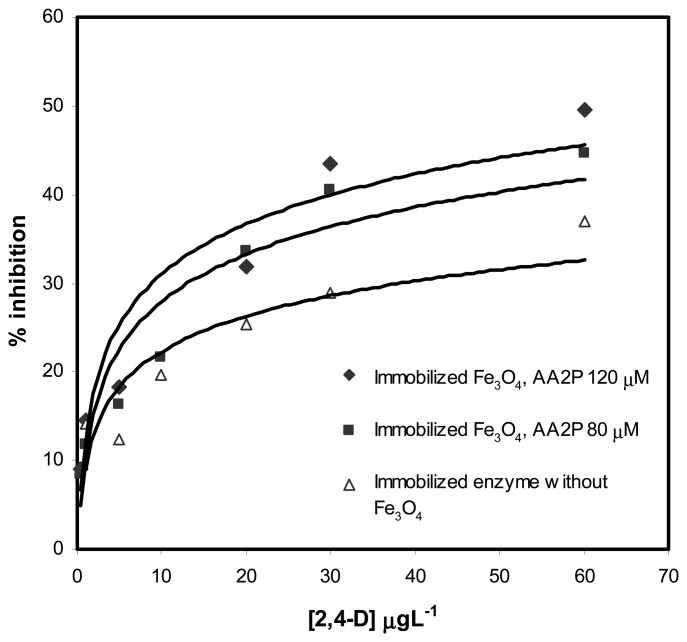
The 2,4-D inhibition profile of the biosensors with and without immobilized Fe_3_O_4_ nanoparticles obtained at two different concentrations of AA2P substrate in 0.1 M Tris-HCl buffer at pH=8.5, applied potential was 0.6 V versus Ag/AgCl reference.

The inhibition of the response by 2,4-D was examined for biosensors with and without immobilized Fe_3_O_4_ nanoparticles. The substrate AA2P was used to evaluate the degree of inhibition after the biosensors were exposed to 2,4-D for 15 min. The percentage of inhibition by 2,4-D on the response for biosensors containing immobilized Fe_3_O_4_ nanoparticles was similar for both 80 and 120 μM of AA2P. But for biosensor without Fe_3_O_4_ nanoparticles, a much reduced response to 80 μM of AA2P was observed (Figure 6).

For biosensors with different added amounts of Fe_3_O_4_ nanoparticles, the percentage of inhibition was approximately 40 % at 30 μgL^-1^ of 2,4-D. The linear response ranges were from 0.5 – 30.0 μgL^-1^ 2,4-D and the detection limits for 2,4-D were from 0.3-0.4 μgL^-1^ (Table 2). For the biosensor without added Fe_3_O_4_ nanoparticles, the maximum inhibition achieved was only 30 % at 30 μg/L of 2,4-D with a slightly shorter linear response range of 1.0-30.0 μgL^-1^ and a detection limit of 0.8 μgL^-1^ of 2,4-D (Figure 5). Based on the linear response range, obviously biosensors with immobilized Fe_3_O_4_ nanoparticles exhibited sensitivity that was almost two times higher than biosensor without Fe_3_O_4_ nanoparticles (Table 2). Approximately 50% improvement in detection limit was also demonstrated when nanoparticles were added. This improved performance of the 2,4-D biosensor might be the results of improved transduction of the inhibition event in the presence of Fe_3_O_4_ nanoparticles.

**Figure 7.**
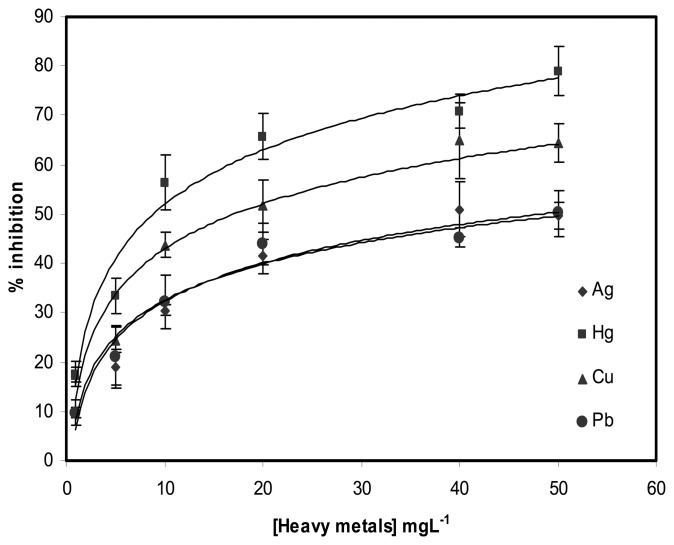
The profiles of inhibition of the biosensor response by several metal ions at 120 μM of AA2P substrate in 0.1 M tris-HCl buffer, pH=8.5, applied potential 0.6 V versus Ag/AgCl reference. Fe_3_O_4_ nanoparticles immobilized was 0.4 wt%.

The inhibition profiles of the biosensor response by various heavy metals (Hg^2+^, Cu^2+^, Ag^2±^and Pb^2+^) is shown in Figure 7. Among the metal ions tested, Hg^2+^ caused the highest inhibition effect, which was approximately 70 % at 40 mgL^-1^. The inhibition of the biosensor response caused by other heavy metals was 65% for Cu^2+^; 51% for Ag^2+^ and 45% for Pb^2+^ at the same concentration. Therefore, the degree of inhibition is Hg^2+^ > Cu^2+^ > Ag^2+^ > Pb^2+^ and this is similar to that reported by Sanchez et al. [23] who had used the free and immobilized ALP enzyme for the detection of heavy metals. The inhibition of the biosensor response caused by these heavy metals was observed at much higher concentrations when compared with inhibition that was caused by 2,4-D alone, which is typically of concentration 1000 times lower. This indicates that the biosensor has higher sensitivity towards 2,4-D when compared to heavy metals.

### 2.3 The analytical performance of biosensor for 2,4-D determination

Water from a rice field was used as a test media for the recovery study because 2,4-D is often used in rice fields to control weed growth. The average percentages of recovery from spiking the water samples with 10 and 20 μgL^-1^ of 2,4-D are shown in Table 3. In general, the recovery of 2,4-D as determined from biosensors doped with Fe_3_O_4_ nanoparticles are at acceptable levels of 100.8 ± 20.2% and 95.41 ± 6.15% for 10 and 20 μgL^-1^ of 2,4-D, respectively. These high recovery values implied that the biosensor performance was unaffected by the matrix of the water samples from the rice fields.

**Figure 8.**
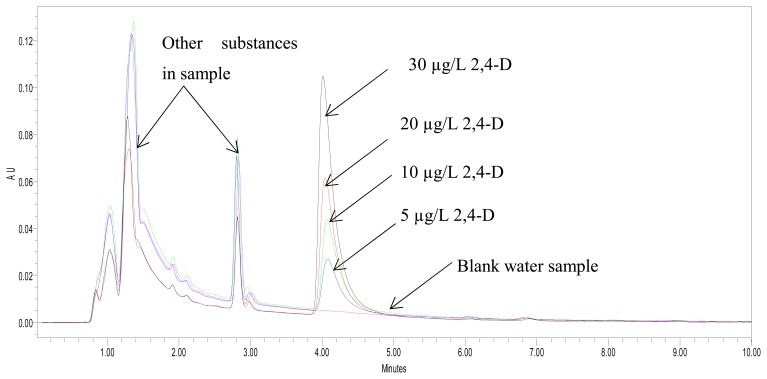
A HPLC chromatogram of water samples from rice fields spiked with 2,4-D standards (5-30 μgL^-1^). The appearance of many other chromatographic peaks indicated that the water sample had a complex matrix.

The performance of the biosensor for the analysis of 2,4-D was further compared with the results of analysis using a HPLC method. The HPLC chromatograms (Figure 8) showed that apart from the spiked 2,4-D, the water samples also contained other substances, which indicated that the matrix of the water samples from the rice field was complex.

Even though the samples had a complex matrix, there was good correlation between the biosensor and HPLC methods for the analysis of 2,4-D (Table 2). Therefore, the biosensor constructed from doping of Fe_3_O_4_ nanoparticles into a sol gel/chitosan membrane containing immobilized ALP has good analytical performance for the determination of 2,4-D.

## 3. Experimental Section

### 3.1 Reagents

Materials used in this research were tetraethoxyorthosilane (TEOS) and ammonium hydroxide purchased from Fluka; hydrochloric acid and acetic acid glacial from Merck. Chitosan, alkaline phophatase (ALP), ascorbic acid-2-phosphate (AA2P), 2,4-dichlorophenoxyacetic acid (2,4-D) and MgCl_2_ were supplied by Sigma and tris(hydroxylmethyl)aminomethane (Tris-HCl) by Ducheta Biochemie. FeCl_3_·6H_2_O and FeCl_2_·4H_2_O were obtained from Erba Pure Reagents. All solutions were prepared in distilled and deionized water. The metal ion solutions were prepared in distilled-deionized water using HgNO_3_ (Merck), AgNO_3_ (Ajax Chemical), PbNO_3_ (General Purpose Reagent), and CuSO_4_ (BDH Laboratory Supplies).

### 3.2 Apparatus and measurement

The chronoamperometric measurements were performed with an AUTOLAB potentiostat with the PG12 (AUT 71681) program. This method was used to measure the current associated with oxidation or reduction of an electroactive species such as AA2P that was involved in the enzyme-substrate reaction with ALP. Amperometric transduction is chosen because it provides high sensitivity and wide linear response range to the biosensor. Screen-printed carbon paste electrodes (SPE) manufactured by Scrint Co was used as the working electrode. The amperometric measurements were performed at 0.6 V versus Ag/AgCl as the reference electrode, and a platinum electrode as the auxiliary electrode. All experiments were performed in 0.1M Tris-HCl buffer (pH 8.5) containing 1.0 mM MgCl_2_. The solution was stirred during amperometric measurement. For HPLC analysis, a Waters 1525 instrument equipped with a Waters 2487 dual λ spectrophotometric detector was used. Strata X cartridges (Phenomenex) were used for the extraction procedure before HPLC analysis was carried out.

### 3.3 Preparation and characterization of Fe_3_O_4_ nanoparticles

The magnetic nanoparticles, Fe_3_O_4_ were synthesized using Fe^2+^ and Fe^3+^ via chemical precipitating with NH_4_OH and treating under hydrothermal conditions [30]. The ferric and ferrous chlorides with the molar ratio 2:1 were dissolved in water at a concentration of 0.3M. This solution was stirred, followed by slow addition of 30% NH_4_OH solution. The color of the solution changed from orange to black and finally produced a black precipitate. The precipitates were then heated at 80°C for 30 min followed by washing several times with water and ethanol. Finally, it was dried in oven at 70°C. The structural properties of Fe_3_O_4_ nanoparticles were analyzed by an X-ray diffractometer (Siemen, Model D-5000). The morphology, particle size and structure of the Fe_3_O_4_ nanoparticles were determined by a Philips CM12 transmission electron microscopy (TEM) operating at 100 keV.

### 3.4 Preparation of electrodes/biosensor

To prepare the sol-gel/chitosan hybrid membrane for immobilization of enzyme ALP and Fe_3_O_4_ nanoparticles, a sol-gel solution containing TEOS (2 mL), deionized water (3 mL) and 1.0 M HCl (0.10 mL) was prepared in a glass vial and stirred for an hour. A 1 wt% chitosan solution was prepared in 1 wt% acetic acid and stirred to dissolve overnight. The solution of hybrid sol-gel/ chitosan was prepared by mixing both the sol-gel and chitosan solutions in the ratio of 9:1 (w/w). The sol-gel/chitosan solution was then mixed with certain amount of Fe_3_O_4_ nanoparticles and enzyme alkaline phosphatase (ALP) in a vial. To fabricate the biosensor, sol-gel/chitosan solution (5 μL) with ALP/Fe_3_O_4_ (1 Unit ALP and 0.4 wt% or 2.2 wt% Fe_3_O_4_) was drop-coated onto the surface of a SPE and allowed to dry overnight. A thin layer of film containing physically entrapped ALP/Fe_3_O_4_ was formed. These electrodes were then used for the investigation of the electrochemical behavior of the immobilized ALP and Fe_3_O_4_ and inhibition studies of 2,4-D and heavy metals.

### 3.5 Optimization of biosensor response

#### Optimization of applied potential

The SPE coated with the biosensor film was connected to an Ag/AgCl electrode as the reference electrode and Pt as the auxiliary electrode. The SPE containing the biosensor membrane was conditioned with tris-HCl buffer (20 μL) for a fixed duration of 15 min. During conditioning, the polymer membrane would be hydrated and this would facilitate the diffusion of analyte into the membrane when the electrode was exposed to the substrate. The electrode was later immersed in Tris-HCl buffer (0.1 M, pH 8.5) containing 1 mM MgCl_2_ and 80 μM substrate AA2P for a fixed time of 5 min before they were scanned in a potential range of 0.3-0.8 V in order to establish the optimum operation potential for the biosensor. The same procedure was repeated using a SPE coated with only ALP/sol-gel/chitosan membrane (no Fe_3_O_4_ nanoparticles). The maximum current response observed at a particular applied potential was later employed for further experiments.

#### Optimization of pH

The working pH for the biosensor was optimized by measuring the current response of the biosensor in buffer solutions (0.1M) of pH ranged from 4.0-10.5. The applied potential used was at 0.6 V and substrate AA2P concentration was fixed at 80 μM.

#### Optimization of AA2P substrate

The optimization of AA2P substrate concentration was performed in Tris-HCl buffer (0.1 M, pH 8.5) using several concentrations at 5 to 120 μM. The current response was recorded with each AA2P concentrations and both biosensors with (0.4 and 2.2 wt%) and without Fe_3_O_4_ nanoparticles were studied.

### 3.6 Inhibition of ALP enzyme by 2,4-D and heavy metals

The inhibition of the biosensor response was investigated by exposing the biosensors to 2,4-D standard solution (0.5 - 60.0 μgL^-1^) or metal ion solutions (1.0-50.0 mgL^-1^ of Hg^2+^, Cu^2+^, Ag^2+^ and Pb^2+^) for 15 min. The inhibition of response can be evaluated by calculating the differences in current before and after exposure to the toxicants. The percentage of inhibition (% I) was calculated as follows:

$$\%\text{Inhibition}\;(\%I)=\frac{I_{O}-I_{A}}{I_{O}}\times 100\%$$

*I_O_* is the current measured for the biosensor before inhibition and *I_A_* is the current after inhibition. The response of the biosensor after inhibition by 2,4-D was evaluated using two substrate concentrations, i.e. 80 and 120 μM AA2P.

### 3.7 Sample preparation for recovery studies

Water samples were taken from rice fields in the agricultural areas of Tanjung Karang (Selangor, Malaysia). The water samples were filtered through a paper filter (Whatman 5, diameter 5 cm, pore size 0.45 μm) to remove the particulate matter and then stored at 4 °C. Recovery tests for 2,4-D were carried out using biosensors doped with Fe_3_O_4_ nanoparticles. For evaluating the recovery of 2,4-D from samples, concentrations of 10 and 20 μgL^-1^ of 2,4-D standards were spiked into the water samples. Recovery percentage of 2,4-D was then calculated after determination with the biosensors.

### 3.8. Comparison of analytical performance of 2,4-D biosensor with HPLC method

In order to compare the analytical performance of the biosensor based on immobilized ALP and Fe_3_O_4_ nanoparticles, water samples from rice fields were spiked with 2,4-D before analysis using a HPLC method. The spiked concentrations of 2,4-D were 5 - 30 μgL^-1^. For the analysis using HPLC method, the spiked samples were first extracted using solid phase extraction with Strata X cartridges (6 mL, 200 mg) according to the procedure of D'Archiovio *et al.* [31]. The cartridges were initially conditioned by washing with dichloromethane (10 mL), followed by acetonitrile (10 mL) and distilled-deionized water (10 mL). The solvents were allowed to flow through the cartridge at a rate of 1 mL min^-1^under a moderate vacuum. A water sample (0.5 L) was then passed through the cartridge at a flow rate of 10 mL min^-1^. Finally, the cartridge was washed with a water-methanol mixture (95:5 v/v) and dried for approximately 10 min by fluxing air. The 2,4-D retained in the cartridge was eluted by 5 mL of acetonitrile and 5 mL of methanol. The eluent was evaporated by a rotavapor (Buchi R-124) at 40 °C. The dry residue obtained was then dissolved in 2.0 mL of water-acetonitrile mixture similar in composition to the HPLC mobile phase (50:50, v/v). For chromatographic analysis, 20 μL of this final extract was used. The column used was a 3.9 mm × 150 mm Symmetry^®^ C18 (Waters) with 5 μm particle size. The system was controlled by the Breeze software. The HPLC analyses were carried out at room temperature using a mixture of acetonitrile and deionized water (acidified with 0.1 % H_3_PO_4_) at the ratio 50:50 (v/v) as a mobile phase. A constant flow rate of 1 mL min^-1^ was used during the analysis. The UV spectra of 2,4-D were recorded at the wavelength of 210 nm. The instrument was first calibrated with 2,4-D standards in the concentration range of 0.08 -200 μgL^-1^.

## 4. Conclusions

In this work, Fe_3_O_4_ nanoparticles have been synthesized and successfully used to enhance the response of a biosensor based on alkaline phosphatase. By utilizing the inhibition properties of the enzyme ALP, the biosensor was shown to be useful for the detection of the herbicide 2,4-D without major interferences from several other toxic metals. The incorporation Fe_3_O_4_ nanoparticles into the biosensor membrane has resulted in a two-fold increase in sensitivity of the biosensor to 2.4-D and a much improved linear response range and detection limit when compared with biosensor where no Fe_3_O_4_ nanoparticles was incorporated. The detection range of the biosensor for 2,4-D was 0.5-30 μgL^-1^ with recovery of 95-100 percent of 2,4-D. The analytical results demonstrated by the biosensor also correlated well with a HPLC method.

## Figures and Tables

**Table 1.** The calibration curves of the biosensor with and without immobilized Fe_3_O_4_ nanoparticles in the presence of various concentrations of AA2P.

**Amount of Fe_3_O_4_(wt %)**	**Slope (nA mM**^−^**^1^)**	**Intercept**	**Correlation coefficient, R^2^**	**Linear range AA2P(μM)**
0	0.606±0.058	6.95±2.58	0.9733	5 - 80
0.4	0.867±0.036	12.86±2.50	0.9915	5 - 120
2.2	0.742±0.024	10.51±2.06	0.9935	5 – 150

**Table 2.** The calibration curves of the biosensor with 0.4 wt% and 0 wt% immobilized Fe_3_O_4_ nanoparticles in the presence of various concentrations of 2,4-D. The inhibition was evaluated in the presence of two concentrations of AA2P substrate.

**Amount of Fe_3_O_4_ (wt %)**	**Concentration of AA2P(μM)**	**Slope (% inhibition/μgL**^−^**^1^)**	**Intercept**	**Correlation coefficient, R^2^**	**Linear range 2,4-D (μgL**^−^**^1^)**
0	80	0.579±0.095	12.41±1.60	0.9255	1 - 30
0.4	80	1.067±0.069	10.24+1.07	0.9834	0.5 - 30
0.4	120	1.041±0.183	13.24±2.83	0.8900	0.5 – 30

**Table 3.** Average recovery values for 2,4-D spiked in the water samples from rice fields.

**Actual amountof 2,4-D spiked (μgL**^−^**^1^)**	**Amount 2,4-D recovered (μgL**^−^**^1^)**	**Recovery (%)**	**Average recovery (%) (n=5)**
10	13.41	134.10	100.84 ± 20.2
	9.61	96.08	
	9.04	90.45	
	8.09	80.85	
	10.27	102.73	
			
20	19.11	95.55	95.41 ± 6.15
	17.08	85.41	
	20.44	102.19	
	19.48	97.42	
	19.29	96.46	
